# The Association of Firearm Caliber With Likelihood of Death From Gunshot Injury in Criminal Assaults

**DOI:** 10.1001/jamanetworkopen.2018.0833

**Published:** 2018-07-27

**Authors:** Anthony A. Braga, Philip J. Cook

**Affiliations:** 1 School of Criminology and Criminal Justice, Northeastern University, Boston, Massachusetts; 2 Sanford Institute of Public Policy, Duke University, Durham, North Carolina

## Abstract

**Importance:**

A foundational issue in firearms policy has been whether the type of weapon used in an assault affects the likelihood of death.

**Objective:**

To determine whether the likelihood of death from gunshot wounds inflicted in criminal assaults is associated with the power of the assailant’s firearm as indicated by its caliber.

**Design, Setting, and Participants:**

Cross-sectional study with multivariate analysis of data on shooting cases extracted by the authors from police investigation files for assaults that took place in Boston, Massachusetts, between January 1, 2010, and December 31, 2014. These data were analyzed between October 1, 2017, and February 18, 2018. In all cases the victim sustained 1 or more gunshot wounds in circumstances that the Boston Police Department deemed criminal. The working sample included all 221 gun homicides and a stratified random sample of 300 nonfatal cases drawn from the 1012 that occurred during the 5-year period. Seven nonfatal cases were omitted because they had been misclassified.

**Exposures:**

The primary source of variation was the caliber of the firearm used to shoot the victim.

**Main Outcomes and Measures:**

Whether the victim died from the gunshot wound(s).

**Results:**

The final sample of 511 gunshot victims and survivors (n = 220 fatal; n = 291 nonfatal) was predominantly male (n = 470 [92.2%]), black (n = 413 [80.8%]) or Hispanic (n = 69 [13.5%]), and young (mean [SD] age, 26.8 [9.4] years). Police investigations determined firearm caliber in 184 nonfatal cases (63.2%) and 183 fatal cases (83.2%). These 367 cases were divided into 3 groups by caliber: small (.22, .25, and .32), medium (.38, .380, and 9 mm), or large (.357 magnum, .40, .44 magnum, .45, 10 mm, and 7.62 × 39 mm). Firearm caliber had no systematic association with the number of wounds, the location of wounds, circumstances of the assault, or victim characteristics, as demonstrated by χ^2^ tests of each cluster of variables and by a comprehensive multinomial logit analysis. A logit analysis of the likelihood of death found that compared with small-caliber cases, medium caliber had an odds ratio of 2.25 (95% CI, 1.37-3.70; *P* = .001) and large caliber had an odds ratio of 4.54 (95% CI, 2.37-8.70; *P* < .001). Based on a simulation using the logit equation, replacing the medium- and large-caliber guns with small-caliber guns would have reduced gun homicides by 39.5%.

**Conclusions and Relevance:**

Firearms caliber was associated with the likelihood of death from gunshot wounds in criminal assault. Shootings with larger-caliber handguns were more deadly but no more sustained or accurate than shootings with smaller-caliber handguns. This conclusion is of direct relevance to the design of gun policy.

## Key Points

**Question:**

Is there an association between the likelihood of death for firearms shooting victims and the caliber of the firearm?

**Findings:**

A cross-sectional study using 5 years of data extracted from investigation files kept by the Boston Police Department determined that the case-fatality rates of assaults inflicting gunshot injury increased significantly with the caliber of the firearm. Caliber was not significantly correlated with other observable characteristics of the assault, including indicators of intent and determination to kill.

**Meaning:**

The findings are foundational to the debate over whether deadly weapons should be better regulated and provide evidence against the common view that whether the victim lives or dies is determined largely by the assailant’s intent and not the type of weapon.

## Teaser

This cross-sectional study uses data from police department investigation files to characterize the association between firearm caliber and likelihood of death in victims and survivors of shootings in Boston, Massachusetts, between 2010 and 2014.

## Introduction

In 1 of 6 cases of criminal assault inflicting a gunshot wound, the victim dies.^1^ Whether any such shooting proves fatal is determined by the number and location of wounds and by the size and weight of the bullets, among other variables.^2,3^ Systematic studies of gunshot wound severity have documented trends associated with the mix of firearms commonly used in assaults.^4,5^ Of particular concern in the 1980s and early 1990s was the rapid shift from revolvers to semiautomatic pistols and from smaller caliber to medium and larger caliber.^6,7^ The pistols were more powerful and enabled the shooter to fire a large number of rounds rapidly, increasing the chance of multiple wounds.^6,7,8^ While speculative, recent studies suggest a continuing trend toward greater wound severity associated with the greater power of firearms in common use.^9,10^ (Improved trauma care may have prevented an increase in the national case-fatality rate.^11^) It is widely accepted among medical and public health professionals that the likelihood of death in an assault increases with the power of the gun.^2,3,6,7,8,12^ But that belief is routinely challenged by advocates and some social scientists in the national debate over gun regulation.^13,14^

The opposing view holds that it is not the type of weapon that determines whether the victim lives or dies, but rather the intent of the assailant.^13,14,15,16,17^ This notion is captured by the old slogan “guns don’t kill people; people kill people.” In this view, an assailant who is determined to kill will do what is necessary to accomplish that purpose, regardless of weapon type.^11,12,13,14,15,16,17,18,19^ As a logical corollary, the 1 in 6 who die from a gun assault differ from those who survive with respect to the shooter’s intent and determination. In effect, the criminal law and the courts conform with this view by treating a fatal shooting as a more serious crime than a nonfatal shooting. The outcome of the shooting (life or death) is viewed as a reliable guide to the intent—the determination to kill—of the shooter. The most severe punishments, including the death penalty and life in prison, are reserved for cases in which the victim dies.

In 1972, Franklin Zimring published a seminal article that challenged the belief that the outcome of the shooting was primarily determined by the intent of the shooter.^20^ He found that nonfatal and fatal shootings were very similar with respect to the circumstances and observed characteristics of the victims and assailants. In effect, the survivors were “lucky” in that in many cases a small change in the path of the bullet would have resulted in the victim’s death. A notable pattern in Zimring’s data was that the likelihood of death was correlated with the caliber of the assailant’s firearm. He concluded that the outcome of gun assaults had a large random element, and that the power of the firearm was one systematic factor influencing the likelihood that an individual with a gunshot injury would survive, a phenomenon he dubbed “instrumentality.” The contrary view is that the caliber of the gun is simply a reflection of the assailant’s determination to kill, with little independent influence on the probability of death.^11,12,13,14,15,16,17,18,19^

The importance of instrumentality in the gun debate is illustrated by findings from a recent survey of experts by the RAND Corporation. Respondents were asked to estimate the effect on the overall homicide rate of a policy that was successful in reducing the firearms homicide rate. Those respondents who on other items had favored permissive firearms regulation tended to believe that assailants would substitute other weapons with nearly the same effect, unlike those respondents who favored more restrictive regulations: “Median responses by the permissive class suggested that 90 percent of prevented firearm homicides would end as a homicide by another means, and median responses by the restrictive class estimated that just 20 percent would. The middle 50 percent of responses from each group (ie, between the 25th and 75th percentile) did not overlap.”^21^

The relative importance of chance vs intention in determining the likelihood of death cannot be measured because there is no direct measure of intention in available data. But if Zimring is correct that the caliber of the gun in an assault is not correlated with systematic factors such as the skill and determination of the assailant, then the pattern of case-fatality rates across calibers provides a clean test of instrumentality, akin to an experiment.

This study follows the original Zimring analysis but with better data and more sophisticated statistical techniques.

## Methods

Official incident reports for 221 homicides and 1012 nonfatal gun assaults where victims and survivors sustained gunshot wounds were accessed through the Boston Police Department (BPD). These represent all cases known to the BPD for the period January 1, 2010, to December 31, 2014, that were deemed to be criminal by the BPD (not justified or self-inflicted). The research team did not have the resources to code all of the nonfatal cases, and instead selected a stratified random sample of 300 gunshot survivors by randomly selecting 60 survivors per year. Of the selected cases, 1 was excluded because the event did not occur in the BPD’s jurisdiction, and 6 were excluded because it was determined that the individual had not been shot. One gun homicide and 2 nonfatal assaults were committed with shotguns and excluded from the study because of the difficulty of comparing the type of wounds generated by the multiple pellets of a shotgun blast with those created by bullets. The final nonfatal shooting survivor sample included 291 individuals and the final fatal shooting victim sample included 220 individuals.

The institutional review board at Northeastern University approved the study with the requirement that gunshot victim and survivor identities were kept confidential by the removal of all personal identifying information (informed consent from the participants was not required). The study conforms with the Strengthening the Reporting of Observational Studies in Epidemiology (STROBE) reporting guideline for cross-sectional research studies.^22^

The research team attempted to acquire detailed information on the 511 fatal and nonfatal shootings by interviewing investigators and reviewing incident reports and detective case files (including emergency medical response and coroner reports). The case files for the homicide victims were generally complete in recording number and location of wounds, but some of the files for nonfatal cases were missing this information. Furthermore, some homicide and assault cases were missing data on firearm caliber, either because cartridge and bullet fragments were not recovered, or if recovered were too damaged to make a determination of the specific caliber. For analyses requiring data on caliber, the working sample included 367 cases (184 nonfatal and 183 fatal). There were no systematic differences between cases included in the analysis and those excluded because of absence of caliber data (eTable 1 in the Supplement).

### Statistical Analysis

Descriptive statistics were used to compare the characteristics of fatal and nonfatal criminal shootings with respect to the demographic characteristics of gunshot victims and survivors, the circumstances of the assault, and other variables. Multivariate binary and multinomial logistic regression models were used to estimate the relationship between a categorical outcome variable and a predictor variable, holding the influence of other variables constant. The conventional 2-tailed .05 level of significance was selected as the benchmark to reject the null hypothesis of no difference in the association between variables. Missing data for caliber and wounds were analyzed using multivariate logistic regression to ascertain if missingness was systematic or as good as random (eTable 2 in the Supplement).

The primary analysis, which uses the working sample, had 3 goals. The first goal was to determine whether caliber is statistically independent of observable characteristics of the assault, including indicators of skill and intent, to validate the claim that caliber serves as a natural experiment for testing instrumentality. We used χ^2^ tests to compare caliber with each cluster of variables (demographic characteristics of the victim, circumstances of the assault, neighborhood in which the assault occurred, whether it was indoors or outdoors, number of shots fired, and number and location of wounds). In addition, a multinomial logit equation was estimated with all these covariates included.

The second goal was to use this natural experiment to estimate the causal relationship between caliber of the assailant’s firearm and likelihood that the victim died. The third goal, building on the second, was to estimate the effect of a hypothetical intervention, namely replacing all the medium- and large-caliber guns with small-caliber guns. Using the logit regression equation on outcome as the basis for the simulation, the probability of death was calculated for each assault (and then averaged) under both the observed calibers (small, medium, and large) and the hypothetical case of replacing all larger calibers with small.^23^ The percentage reduction in fatalities (homicides) was a measure of the overall effect of instrumentality associated with caliber for our sample.

It should be noted that the probabilities of death reflect the fact that the nonfatal shootings are a sample of the total, whereas nearly all homicides are included. Hence the computed probabilities are a multiple of the true probabilities of death. But the ratio of estimated probabilities should be unaffected, as the multiple is the same for the numerator and denominator of the ratio.

In all analyses, caliber was coded as either small (.22, .25, and .32), medium (.38, .380, and 9 mm), or large (.357 magnum, .40, .44 magnum, .45, 10 mm, and 7.62 × 39 mm). The wound location was coded as head or neck; chest, back, or abdomen; or arms and legs. The number of wounds was coded as 1 or more than 1.

Stata SE 14.1 statistical software (StataCorp) was used to calculate the maximum likelihood estimates of the parameters for the predictor variables and to compute the associated probability values. In line with current best practice, robust standard errors were used, clustered by police district. Policing districts were used as indicators for Boston neighborhoods to capture both variations in community contexts and local policing practices that could affect the lethality of shootings and the quality of investigative information.^24,25^

## Results

Table 1 presents the characteristics of gunshot victims and survivors and circumstances in gun assaults and homicides occurring in Boston between January 1, 2010, and December 31, 2014. The final sample of 511 gunshot victims and survivors (220 fatal and 291 nonfatal) was predominantly male (n = 470 [92.2%]), black (n = 413 [80.8%]) or Hispanic (n = 69 [13.5%]), and young (mean [SD] age, 26.8 [9.4] years). The fatal and nonfatal cases have very similar distributions across each variable, including victim and survivor sex, race/ethnicity, age, and criminal history; circumstances that led to the shooting; and police district. For only 1 variable is there a statistically significant difference between fatal and nonfatal cases, and that is whether the shooting occurred indoors or outdoors. Fatal shootings were more likely to occur indoors (54 of 220 [24.5%]) relative to nonfatal shootings (39 of 291 [13.4%]), with a difference of 11.1 percentage points (95% CI, 4.2%-18.0%; *P* = .001). Most gunshot victims and survivors were young minority men with prior court arraignments. Most attacks occurred in circumstances where gangs or drugs played an important role (according to BPD investigation results). Most were in outdoor locations in the disadvantaged Boston neighborhoods of Roxbury, Mattapan, and Dorchester.

**Table 1.** Wounded Individuals, Circumstances, and Locations of Criminal Shootings in Boston, 2010-2014

Characteristic	No. (%)		Test Statistic	*P* Value
	Nonfatal (n = 291)	Fatal (n = 220)		
Sex				
Male	270 (93.1)	200 (90.9)	χ^2^_1_ = 0.854	.36
Female	21 (6.9)	20 (9.1)		
Race				
Black	231 (79.4)	182 (82.7)	χ^2^_3_ = 1.219	.75
Hispanic	41 (14.1)	28 (12.8)		
White	15 (5.2)	8 (3.6)		
Asian or other	4 (1.3)	2 (0.9)		
Age, mean (SD), y	27.0 (9.1)	26.4 (9.6)	*t* = .721	.47
Criminal history				
Prior arraignments, mean (SD), No.	12.4 (11.3)	11.9 (10.7)	*t* = .506	.61
Circumstance^a^				
Gang	166 (66.5)	147 (66.8)	χ^2^_5_ = 2.809	.73
Drug	40 (15.9)	38 (17.1)		
Personal dispute	38 (13.1)	22 (10.1)		
Robbery	6 (2.5)	8 (3.7)		
Domestic	5 (2.0)	4 (1.8)		
Other	0	1 (0.5)		
Location				
Outdoor	252 (86.6)	166 (75.5)	χ^2^_1_ = 10.449	.001
Indoor	39 (13.4)	54 (24.5)		
Police district				
A-1: Downtown	7 (2.4)	4 (1.8)	χ^2^_11_ = 9.434	.58
A-7: East Boston	5 (1.7)	2 (0.9)		
A-15: Charlestown	1 (0.3)	4 (1.8)		
B-2: Roxbury	82 (28.2)	63 (28.6)		
B-3: Mattapan	71 (24.4)	60 (27.2)		
C-6: South Boston	7 (2.4)	5 (2.3)		
C-11: Dorchester	65 (22.3)	34 (15.5)		
D-4: Back Bay, Fenway	15 (5.2)	15 (6.8)		
D-14: Allston, Brighton	4 (1.4)	3 (1.4)		
E-5: West Roxbury	7 (2.4)	10 (4.5)		
E-13: Jamaica Plain	14 (4.8)	11 (5.0)		
E-18: Hyde Park	13 (4.5)	9 (4.1)		

^a^ Circumstance percentages excluded shooting victims and survivors whose assailants had unknown motives.

Table 2 reports the distribution of calibers, shots fired, number of gunshot wounds, and wound location for nonfatal and fatal cases. Police investigations determined firearm caliber in 184 nonfatal cases (63.2%) and 183 fatal cases (83.2%). Most interpersonal gun violence involves handguns, and Boston is no exception. Only 1 gun homicide was committed with a rifle caliber (7.62 × 39 mm fired from an AK-47 assault rifle). The most common caliber was 9 mm in both nonfatal shootings (50 of 184 [27.2%]) and gun homicides (65 of 183 [35.6%]). Homicides were more likely to involve large-caliber firearms (60 of 183 [32.8%]) relative to nonfatal shootings (33 of 184 [17.9%]).

**Table 2.** Firearm Calibers and Wounds for Criminal Shootings in Boston, 2010-2014

Characteristic	No. (%)	
	Nonfatal (n = 184)	Fatal (n = 183)
Caliber		
.22	14 (7.6)	6 (3.3)
.25	11 (6.0)	5 (2.7)
.32	13 (7.1)	12 (6.6)
.380	32 (17.4)	17 (9.3)
.38	31 (16.8)	18 (9.8)
9 mm	50 (27.2)	65 (35.5)
.357 Magnum	5 (2.7)	13 (7.1)
.40	15 (8.2)	18 (9.8)
.44 Magnum	1 (0.5)	2 (1.1)
.45	12 (6.5)	24 (13.1)
10 mm	0	2 (1.1)
7.62 × 39 mm	0	1 (0.6)
Small caliber	38 (20.7)	23 (12.6)
Medium caliber	113 (61.4)	100 (54.6)
Large caliber	33 (17.9)	60 (32.8)
Shots fired, mean (SD), No.	4.41 (3.98)	6.11 (5.73)
No. of wounds		
Single	134 (72.8)	64 (35.0)
Multiple	50 (27.2)	119 (65.0)
Mean (SD)	1.67 (1.41)	2.82 (2.76)
Single-wound location		
Total single wounds	134 (100)	64 (100)
Head, neck	9 (6.7)	35 (54.7)
Chest, back, abdomen	41 (30.6)	28 (43.8)
Arms, shoulders, legs	84 (62.7)	1 (1.6)
Multiple-wound location		
Total multiple wounds	50 (100)	119 (100)
Head, neck	6 (12.0)	60 (50.4)
Chest, back, abdomen	26 (52.0)	58 (48.7)
Arms, shoulders, legs	18 (36.0)	1 (0.8)

The number of shots fired in each case was estimated by the BPD based on spent bullets and cartridge casings recovered at the crime scene. The mean (SD) number of shots was higher in fatal shootings (6.11 [5.73]) than in nonfatal shootings (4.41 [3.98]). Homicide victims were more likely to have multiple gunshot wounds (119 of 183 [65.0%]) than were nonfatal shooting survivors (50 of 184 [27.2%]), and the mean (SD) number of gunshot wounds for homicide victims was correspondingly higher than the number for survivors (2.82 [2.76] vs 1.67 [1.41], respectively). A separate calculation found that the number of shots fired was statistically unrelated to caliber for both fatal and nonfatal cases (eTable 3 in the Supplement).

The distributions of wound locations differed in the expected way. For individuals with a single wound, 84 of 134 (62.7%) were peripheral (legs, arms, or shoulders) for nonfatal cases, compared with only 1 of 64 (1.6%) for fatal cases. For individuals with multiple wounds, the most serious wound was peripheral in 18 of 50 nonfatal shootings (36.0%) compared with 1 of 119 fatal shootings (0.8%). (The ranking used to determine seriousness was based only on location, with head and neck most serious; then chest, back, and abdomen; then arms, shoulders, and legs.

Table 3 provides results relevant to the first goal of the analysis. It presents the results of a multinomial logistic regression on the caliber of the gun used in the assault. In model 1, the variables include the sex, race, and age of the victim or survivor, the circumstances (motivation) of the attack, and whether the attack occurred indoors or outdoors. Model 2 adds additional covariates, including the number of wounds and location of the most serious wounds (both considered indicators of skill and determination to kill). There are no statistically significant results for either model. Supplemental χ^2^ analyses of association of caliber with victim or survivor and shooting characteristics also yielded statistically nonsignificant results, confirming the results of the multivariate analysis (eTable 4 in the Supplement). The lack of systematic association is what would be expected if caliber were assigned at random, as in an experiment.

**Table 3.** Multinomial Logistic Regressions of Shooting Characteristics on Caliber

Characteristic^a^	Logistic Regression Coefficient (Robust Standard Error)			
	Model 1^b^		Model 2^b^	
	Large Caliber	Medium Caliber	Large Caliber	Medium Caliber
Sex (male)				
Female	1.103 (0.809)	0.886 (0.748)	1.231 (0.926)	0.978 (0.744)
Race (white)				
Black	0.087 (0.847)	0.504 (0.704)	0.204 (0.765)	0.641 (0.812)
Hispanic	0.332 (0.947)	0.246 (0.791)	0.638 (0.903)	0.827 (0.895)
Asian or other	0.937 (1.051)	0.871 (0.687)	0.886 (0.993)	0.415 (0.711)
Age	−0.008 (0.023)	0.007 (0.019)	−0.010 (0.024)	−0.002 (0.021)
Prior arraignments	−0.018 (0.013)	−0.011 (0.012)	−0.013 (0.015)	−0.013 (0.013)
Circumstances (other)				
Gang	0.981 (0.809)	0.897 (0.490)	0.988 (0.734)	0.768 (0.531)
Drug	0.876 (0.719)	0.708 (0.607)	0.814 (0.896)	0.735 (0.660)
Personal dispute	0.493 (0.782)	0.787 (0.621)	1.106 (0.925)	0.861 (0.673)
Robbery	−0.506 (1.290)	0.332 (0.846)	0.084 (1.321)	0.715 (0.855)
Domestic	0.495 (0.337)	0.341 (0.239)	0.509 (0.357)	0.309 (0.231)
No. of wounds (single wound)				
Multiple wounds	NI	NI	0.209 (0.383)	0.167 (0.325)
Most serious wound location (leg, arm, shoulder)				
Chest, back, abdomen	NI	NI	−0.609 (0.484)	−0.597 (0.405)
Head, neck	NI	NI	0.791 (0.569)	0.119 (0.505)
Scene location (outdoor)				
Indoor	0.049 (0.462)	0.051 (0.399)	−0.037 (0.475)	0.086 (0.402)
Shots fired	NI	NI	0.007 (0.040)	0.011 (0.036)
Constant	−0.431 (1.215)	−0.0242 (0.953)	−0.988 (1.295)	−0.207 (1.075)
Log pseudolikelihood	−339.052		−311.541	
Pseudo *R*^2^	0.037		0.062	
Individuals included in analysis, No.	344		344	

Abbreviation: NI, not included.

^a^ The default categories for the dummy variables are identified in parentheses.

^b^ Base category in the multinomial dependent variable is small caliber.

Table 4 presents the results of a multivariate logistic regression on whether the event was fatal or nonfatal. The effects of caliber on odds of death are estimated, controlling for gunshot victim and survivor characteristics, whether the shooting occurred indoors or outdoors, and neighborhood indicators (included, not shown). Relative to shootings involving small-caliber firearms (reference category), the odds of death if the gun was large caliber were 4.5 times higher (OR, 4.54; 95% CI, 2.37-8.70; *P* < .001) and, if medium caliber, 2.3 times higher (OR, 2.25; 95% CI, 1.37-3.70; *P* = .001). The odds of death in indoor shootings were 2.6 times higher than in outdoor shootings (OR, 2.55; 95% CI, 1.79-3.64; *P* < .001). The effects of caliber size and indoor location remained strong in the alternate specifications in models 2 and 3. None of the other covariates had a statistically discernible effect on the odds of death.

**Table 4.** Multivariate Logistic Regressions of Caliber and Event Characteristics on Shooting Outcome

Characteristic^a^	Odds Ratio	Logistic Regression Coefficient (Robust Standard Error)
Caliber (small)		
Medium	2.251	0.811 (0.253)^b^
Large	4.543	1.513 (0.331)^b^
Sex (male)		
Female	1.051	0.051 (0.386)
Race (white)		
Black	1.997	0.691 (0.652)
Hispanic	1.769	0.571 (0.681)
Asian or other	0.876	−0.132 (1.308)
Age	1.000	0.001 (0.009)
Prior arraignments	1.000	0.001 (0.008)
Scene location (outdoor)		
Indoor	2.553	0.937 (0.180)^b^
Constant	−1.910 (0.784)^c^	
Log pseudolikelihood	−324.885	
Pseudo *R*^2^	0.075	
Individuals included in analysis, No.	344	

^a^ The default categories for the dummy variables are identified in parentheses. Area dummy variables are included but not shown in the table. Robust standard errors were clustered by 12 Boston Police Department districts.

^b^ *P* < .01.

^c^ *P* < .05.

The overall effect of larger caliber on deaths can be estimated by simulation using the logit regression results in Table 4. The simulation uses the 367 shooting cases (184 nonfatal [63.2%] and 183 fatal [83.2%]) with known caliber. First, the predicted probability of death for each shooting case was computed using the actual caliber (mean [SD] probability, 0.499 [0.154]), and then the predicted probability on the assumption that all shootings had been with a small-caliber gun (mean [SD] probability, 0.302 [0.137]). The ratio of mean probabilities was 0.605. The implication is that if the medium- and large-caliber guns had been replaced with small caliber (assuming everything else unchanged), the result would have been a 39.5% reduction in gun homicides.

## Discussion

In a pioneering article published in 1972, Franklin Zimring sought to demonstrate that the type of weapon used in a criminal assault had a causal effect on the likelihood that the victim would die.^20^ He compiled a data set that allowed him to compare the case fatality rates for criminal shootings by caliber of weapon, which he asserted was a sort of “natural experiment.” He demonstrated that fatality rate was positively correlated with caliber and argued that this gradient was a direct reflection of the intrinsic power and lethality of the weapon. In this article we revisit his analysis with more complete data and more sophisticated statistical methods. We were able to confirm the strong positive association between caliber and fatality rate and summarize the overall effect of larger calibers by simulating the effect on outcomes if all the guns had been small caliber. The result is a 39.5% reduction in the probability of death, implying an equal reduction in the gun homicide rate if the same shootings had occurred but with small-caliber weapons, rather than the actual mix of small, medium, and large calibers.

That result suggests that the instrumentality effect is large even if the analysis is limited to gun caliber. It would also be of interest to quantify the effect of replacing guns of all types with other commonly available weapons. Presumably the effects would be still larger, although the precise nature of the substitution would have to be specified.

### Limitations

The main challenge to the results in the current analysis is the possibility that shooters who used large-caliber guns, in comparison with those who used smaller-caliber guns, were somehow more determined to kill or more skillful at killing in ways that are not well measured by the number and location of wounds. That possibility is created by the fact that guns are not randomly assigned to shooters. But we demonstrated that just as if there were random assignment, caliber was uncorrelated with all observable aspects of the shootings except whether the wounded person lived or died.

There are other limitations of this study. First, caliber was not available for all shootings. We found no statistical pattern of missingness within each of the 2 categories of fatal and nonfatal, but that does not completely settle the issue. Second, the study is limited to the criminal shootings known to the police in a particular time and place.

## Conclusions

Whether the type of weapon matters in affecting the outcome of a shooting is a foundational issue in the debate over appropriate gun regulation. The results here support the view that the intrinsic power and lethality of the weapon had a direct effect on the likelihood that a victim of a criminal shooting died. For Boston, in the period studied here, simply replacing larger-caliber guns with small-caliber guns with no change in location or number of wounds would have reduced the gun homicide rate by 39.5%. It is plausible that larger reductions would be associated with replacing all types of guns with knives or clubs.

The finding that the type of weapon is associated with fatality rate provides insight into the nature of homicide. Whether the victim of a serious assault lives or dies is to a large extent a matter of chance, rather than a question of the assailant’s intent. The probability of death is connected to the intrinsic power and lethality of the weapon. That suggests that effective regulation of firearms could reduce the homicide rate. That conclusion is relevant to the national debate over gun regulation, although insufficient in itself to demonstrate that any particular regulation would satisfy a cost-benefit test.

## Notes

Supplement. **eTable 1.** Wounded Individual Characteristics, Circumstances, and Locations of Criminal Shootings by Missing and Non-Missing Caliber Data **eTable 2.** Multivariate Logistic Regressions of Missing Wound and Missing Caliber Data for Gun Homicides and Non-Fatal Gun Assaults **eTable 3.** Comparison of Shots Fired by Caliber in Criminal Gun Assaults **eTable 4.** Gunshot Victim and Survivor Characteristics and Shooting Characteristics by Caliber Sizes

## References

[1] Cook PJ, Rivera-Aguirre AE, Cerdá M, Wintemute G Constant lethality of gunshot injuries from firearm assault: United States, 2003-2012. Am J Public Health. 2017;107(8):-. doi:10.2105/AJPH.2017.303837 28640677 PMC5508146

[2] Hargarten SW, Karlson TA, O’Brien M, Hancock J, Quebbeman E Characteristics of firearms involved in fatalities. JAMA. 1996;275(1):42-45. doi:10.1001/jama.1996.03530250046025 8531285

[3] Fackler ML, Malinowski JA The wound profile: a visual method for quantifying gunshot wound components. J Trauma. 1985;25(6):522-529. doi:10.1097/00005373-198506000-00009 4009751

[4] Webster DW, Champion HR, Gainer PS, Sykes L Epidemiologic changes in gunshot wounds in Washington, DC, 1983-1990. Arch Surg. 1992;127(6):694-698. doi:10.1001/archsurg.1992.01420060066010 1596170

[5] McGonigal MD, Cole J, Schwab CW, Kauder DR, Rotondo MF, Angood PB Urban firearm deaths: a five-year perspective. J Trauma. 1993;35(4):532-536. doi:10.1097/00005373-199310000-00006 8411275

[6] D’Alessio JG Gunshot wounds: bullet caliber is increasing. J Trauma. 1999;47(5):992-993. 10568740 10.1097/00005373-199911000-00037

[7] Wintemute GJ The relationship between firearm design and firearm violence: handguns in the 1990s. JAMA. 1996;275(22):1749-1753. doi:10.1001/jama.1996.03530460053031 8637173

[8] Kellermann AL, Lee RK, Mercy JA, Banton J The epidemiologic basis for the prevention of firearm injuries. Annu Rev Public Health. 1991;12:17-40. doi:10.1146/annurev.pu.12.050191.000313 2049134

[9] Sauaia A, Gonzalez E, Moore HB, Bol K, Moore EE Fatality and severity of firearm injuries in a Denver trauma center, 2000-2013. JAMA. 2016;315(22):2465-2467. doi:10.1001/jama.2016.5978 27299622

[10] Livingston DH, Lavery RF, Lopreiato MC, Lavery DF, Passannante MR Unrelenting violence: an analysis of 6,322 gunshot wound patients at a level I trauma center. J Trauma Acute Care Surg. 2014;76(1):2-9. doi:10.1097/TA.0b013e3182ab19e7 24368351

[11] Kent AJ, Sakran JV, Efron DT, Haider AH, Cornwell EE III, Haut ER Understanding increased mortality after gunshot injury. Am J Public Health. 2017;107(12):e22-e23. doi:10.2105/AJPH.2017.304100 29116838 PMC5678393

[12] Karlson TA, Hargarten SW Reducing Firearm Injury and Death: A Public Health Sourcebook on Guns. New Brunswick, NJ: Rutgers University Press; 1997.

[13] Kleck G Point Blank: Guns and Gun Violence in America. Hawthorne, NY: Aldine de Gruyter; 1997.

[14] Blackman PH A critique of the epidemiological study of firearms and homicide. Homicide Stud. 1997;1(2):169-189. doi:10.1177/1088767997001002005

[15] Hahn RA, Bilukha OO, Crosby A, . Task Force on Community Preventive Services First reports evaluating the effectiveness of strategies preventing violence: firearms laws: findings from the task force on community prevention services. MMWR Recomm Rep. 2003;52(RR-14):11-20. 14566221

[16] Institute of Medicine and National Research Council Priorities for Research to Reduce the Threat of Firearm-Related Violence. Washington, DC: National Academies Press; 2013.

[17] National Research Council Firearms and Violence: A Critical Review. Washington, DC: National Academies Press; 2005.

[18] Wolfgang M Patterns in Criminal Homicide. Philadelphia: University of Pennsylvania Press; 1958. doi:10.9783/9781512808728

[19] Wright JD, Rossi PH, Daly K Under the Gun: Weapons, Crime, and Violence in America. Hawthorne, NY: Aldine de Gruyter; 1983.

[20] Zimring FE The medium is the message: firearm caliber as a determinant of death from assault. J Legal Stud. 1972;1:97-124. doi:10.1086/467479

[21] Morral AR, Schell TL, Tankard M The Magnitude and Sources of Disagreement Among Gun Policy Experts. Santa Monica, CA: RAND Corporation; 2018. doi:10.7249/RR2088.1

[22] von Elm E, Altman DG, Egger M, Pocock SJ, Gøtzsche PC, Vandenbroucke JP; STROBE Initiative The Strengthening the Reporting of Observational Studies in Epidemiology (STROBE) statement: guidelines for reporting observational studies. J Clin Epidemiol. 2008;61(4):344-349. doi:10.1016/j.jclinepi.2007.11.008 18313558

[23] Muller CJ, MacLehose RF Estimating predicted probabilities from logistic regression: different methods correspond to different target populations. Int J Epidemiol. 2014;43(3):962-970. doi:10.1093/ije/dyu029 24603316 PMC4052139

[24] Harding DJ Living the Drama: Community, Conflict and Culture Among Inner-City Boys. Chicago, IL: University of Chicago Press; 2010. doi:10.7208/chicago/9780226316666.001.0001

[25] Braga AA, Dusseault D Can homicide detectives improve homicide clearance rates? Crime Delinq. 2018;64(3):283-315. doi:10.1177/0011128716679164

