# Supplementary material for: The Association of Firearm Caliber With Likelihood of Death From Gunshot Injury in Criminal Assaults
Source: JAMA Netw Open. 2018 Jul 27;1(3):e180833. doi: 10.1001/jamanetworkopen.2018.0833 (PMC6324289; doi:10.1001/jamanetworkopen.2018.0833)
Supplement: Supplement. — eTable 1. Wounded Individual Characteristics, Circumstances, and Locations of Criminal Shootings by Missing and Non-Missing Caliber Data eTable 2. Multivariate Logistic Regressions of Missing Wound and Missing Caliber Data for Gun Homicides and Non-Fatal Gun Assaults eTable 3. Comparison of Shots Fired by Caliber in Criminal Gun Assaults eTable 4. Gunshot Victim and Survivor Characteristics and Shooting Characteristics by Caliber Sizes [file jamanetwopen-1-e180833-s001.pdf]

# Supplementary Online Content

Braga AA, Cook PJ. The association of firearm caliber with likelihood of death from gunshot injury in criminal assaults. *JAMA Netw Open*. 2018;1(3):e180833. doi:10.1001/jamanetworkopen.2018.0833

**eTable 1.** Wounded Individual Characteristics, Circumstances, and Locations of Criminal Shootings by Missing and Non-Missing Caliber Data

**eTable 2.** Multivariate Logistic Regressions of Missing Wound and Missing Caliber Data for Gun Homicides and Non-Fatal Gun Assaults

**eTable 3.** Comparison of Shots Fired by Caliber in Criminal Gun Assaults

**eTable 4.** Gunshot Victim and Survivor Characteristics and Shooting Characteristics by Caliber Sizes

This supplementary material has been provided by the authors to give readers additional information about their work.

© 2018 Braga AA et al. *JAMA Network Open.*

eTable 1. Wounded Individual Characteristics, Circumstances, and Locations of Criminal Shootings by Missing and Non-Missing Caliber Data

| | Caliber Present (n = 367) | Caliber Not Present (n = 144) | Test Statistic | *p* |
|---|---|---|---|---|
| *Sex, No. (%)* | | | | |
| Male | 339 (92.4%) | 132 (91.7%) | $Chi^2$ (1) =.071 | .790 |
| Female | 28 (7.6%) | 12 (8.3%) | | |
| | | | | |
| *Race, No. (%)* | | | | |
| Black | 306 (83.4%) | 117 (81.3%) | $Chi^2$ (3) =.684 | .877 |
| Hispanic | 43 (11.7%) | 19 (13.2%) | | |
| White | 14 (3.8%) | 7 (4.9%) | | |
| Asian / other | 4 (1.1%) | 1 (0.7%) | | |
| | | | | |
| *Age* | | | | |
| Years, Mean (SD) | 26.2 (8.63) | 27.3 (10.52) | $\|t\|$ = 1.116 | .266 |
| | | | | |
| *Criminal History* | | | | |
| Prior arraignments, Mean (SD) | 10.65 (12.33) | 10.13 (11.49) | $\|t\|$ =.473 | .637 |
| | | | | |
| *Circumstance, No. (%)* | | | | |
| Gang | 217 (63.8%) | 96 (72.2%) | $Chi^2$ (5) =4.319 | .504 |
| Drug | 57 (16.8%) | 20 (15.0%) | | |
| Personal dispute | 44 (12.9%) | 13 (9.8%) | | |
| Robbery | 13 (3.8%) | 2 (1.5%) | | |
| Domestic | 8 (2.4%) | 2 (1.5%) | | |
| Other | 1 (0.3%) | 0 (0.0%) | | |
| | | | | |
| *Location, No. (%)* | | | | |
| Outdoor | 304 (82.8%) | 116 (80.6%) | $Chi^2$ (1) =.3667 | .545 |
| Indoor | 63 (17.2%) | 28 (19.4%) | | |
| | | | | |
| *Police District, No. (%)* | | | | |
| A-1: Downtown | 14 (3.8%) | 7 (4.9%) | $Chi^2$ (11) =8.476 | .670 |
| A-7: East Boston | 5 (1.4%) | 2 (1.4%) | | |
| A-15: Charlestown | 3 (0.8%) | 2 (1.4%) | | |
| B-2: Roxbury | 96 (26.2%) | 48 (33.3%) | | |
| B-3: Mattapan | 94 (25.6%) | 33 (22.9%) | | |
| C-6: South Boston | 12 (3.3%) | 2 (1.4%) | | |
| C-11: Dorchester | 67 (18.3%) | 30 (20.8%) | | |
| D-4: Back Bay, Fenway | 23 (6.3%) | 7 (4.8%) | | |
| D-14: Allston, Brighton | 5 (1.3%) | 2 (1.4%) | | |
| E-5: West Roxbury | 9 (2.5%) | 3 (2.1%) | | |
| E-13: Jamaica Plain | 22 (6.0%) | 3 (2.1%) | | |

© 2018 Braga AA et al. *JAMA Network Open.*

| | | | | |
|---|---|---|---|---|
| E-18: Hyde Park | 17 (4.6%) | 5 (3.5%) | | |

Note: Circumstance percentages excluded shooting victims and survivors with unknown motives.

eTable 2. Multivariate Logistic Regressions of Missing Wound and Missing Caliber Data for Gun Homicides and Non-Fatal Gun Assaults

| | Model 1 | Model 2 | Model 3 |
|---|---|---|---|
| | Non-Fatal Assault Wounds | Non-Fatal Assault Caliber | Gun Homicide Caliber |
| | Coef. (RSE) | Coef. (RSE) | Coef. (RSE) |
| *Sex (Male)* | | | |
| Female | -.682 (.551) | -.651 (.502) | .610 (.893) |
| | | | |
| *Race (White)* | | | |
| Black | .971 (.721) | -.111 (.607) | 1.006 (.918) |
| Hispanic | .963 (.878) | .189 (.697) | -.1772 (1.032) |
| Asian / other | .117 (.247) | -.315 (.436) | --- |
| | | | |
| Age | -.033 (.027) | -.018 (.013) | -.019 (.018) |
| Prior record | -.001 (.014) | .004 (.011) | .018 (.016) |
| | | | |
| *Circumstances (Other)* | | | |
| Gang | .062 (.557) | .020 (.357) | .113 (.254) |
| Drug | .479 (.764) | .133 (.463) | .741 (1.373) |
| Robbery | -.064 (.982) | 1.297 (.922) | --- |
| Personal dispute | -.571 (.688) | .355 (.587) | .028 (.299) |
| Domestic | --- | 1.060 (1.217) | --- |
| | | | |
| *Scene location (Outdoor)* | | | |
| Indoor | -.069 (.455) | -.529 (.363) | .013 (.477) |
| | | | |
| Constant | 1.988 (1.036) | .902 (.782) | 1.303 (1.641) |
| | | | |
| N | 288 | 293 | 209 |
| Log pseudolikelihood | -109.763 | -185.372 | -91.511 |
| Pseudo $R^2$ | 0.054 | 0.041 | .0765 |

Note: All dependent variables were coded as 0 = data was not present, 1 = data was available. Wounds were known on all N=221 gun homicide victims. Five non-fatal gun assaults with domestic circumstances perfectly predicted the presence of wound data and were dropped from Model 1. One gun homicide involving an Asian victim, 4 gun homicides with domestic circumstances, and 7 gun homicide victims with robbery circumstances perfectly predicted the presence of caliber data and were dropped from Model 3. The default categories for the dummy variables are identified above in parentheses.* $p<.05$, ** $p<.01$

© 2018 Braga AA et al. *JAMA Network Open.*

eTable 3. Comparison of Shots Fired by Caliber in Criminal Gun Assaults

| | N | Mean Shots Fired (SD) |
|---|---|---|
| Small caliber | 61 | 4.82 (4.98) |
| Medium caliber | 215 | 5.30 (5.11) |
| Large caliber | 92 | 5.46 (4.79) |
| | | |
| ANOVA | $F$ = 0.325, $df$ = 2 | $p$=.730 |

Notes: ANOVA = Analysis of Variance, *df* = degrees of freedom, SD = Standard Deviation.

© 2018 Braga AA et al. *JAMA Network Open.*

eTable 4. Gunshot Victim and Survivor Characteristics and Shooting Characteristics by Caliber Sizes

| | Small caliber, No. (%) | Medium caliber, No. (%) | Large caliber, No. (%) | $Chi^2$ *p*-level |
|---|---|---|---|---|
| *Sex (Male)* | | | | |
| Female | 3 (5.1%) | 15 (7.6%) | 8 (9.2%) | .654 |
| | | | | |
| *Race (White)* | | | | .349 |
| Black | 47 (82.5%) | 171 (86.4%) | 71 (79.8%) | .320 |
| Hispanic | 7 (12.3%) | 19 (9.6%) | 14 (15.7%) | .328 |
| Asian / other | 0 (0.0%) | 3 (1.5%) | 0 (0.0%) | |
| | | | | |
| *Age (17 and younger, 31 and older)* | | | | |
| Ages 18 – 30 | 37 (65.2%) | 151 (76.2%) | 59 (66.1%) | .300 |
| | | | | |
| *Criminal History (No priors)* | | | | |
| At least one prior | 47 (82.6%) | 159 (80.2%) | 74 (83.1%) | .894 |
| | | | | |
| *Circumstances (Other)* | | | | |
| Gang | 29 (50.9%) | 124 (62.6%) | 56 (62.9%) | .247 |
| Drug | 9 (15.8%) | 27 (13.6%) | 18 (20.2%) | .365 |
| Personal dispute | 7 (12.3%) | 24 (12.1%) | 7 (7.8%) | .539 |
| Robbery | 3 (5.3%) | 8 (4.0%) | 1 (1.1%) | .334 |
| Domestic | 1 (1.8%) | 3 (1.5%) | 4 (4.5%) | .287 |
| | | | | |
| *Scene location (Outdoor)* | | | | |
| Indoor | 10 (17.5%) | 34 (17.2%) | 17 (19.1%) | .924 |
| | | | | |
| *N wounds (Single wound)* | | | | |
| Multiple wounds | 26 (45.6%) | 97 (49.0%) | 47 (52.8%) | .686 |
| | | | | |
| *Most serious wound location (Leg, arm, shoulder)* | | | | |
| Chest, back, abdomen | 31 (54.4%) | 83 (41.9%) | 36 (40.5%) | .194 |
| Head, neck | 17 (29.8%) | 63 (31.8%) | 37 (41.7%) | .216 |
| | | | | |
| N | 57 | 198 | 89 | |

Note: The default categories for the dummy variables are identified above in parentheses.

© 2018 Braga AA et al. *JAMA Network Open.*
